# Photosynthetic Apparatus of *Hydrocharis morsus-ranae* in Different Solar Lighting

**DOI:** 10.3390/plants11192658

**Published:** 2022-10-10

**Authors:** Elizabeth Kordyum, Oleksandr Polishchuk, Yuri Akimov, Vasyl Brykov

**Affiliations:** 1 Department of Cell Biology and Anatomy, M.G. Kholodny Institute of Botany, National Academy of Sciences of Ukraine, 2 Tereschenkivska Str., 01024 Kyiv, Ukraine; 2 Department of Experimental Plant Biology, Faculty of Science, Charles University, Viničná 5, 128 00 Prague, Czech Republic

**Keywords:** acclimation, anthocyanin, chloroplast, chlorophyll induction, granum, pigment complex, plasticity, principal component analysis, shade, sunlight

## Abstract

*Hydrocharis morsus-ranae* is a free-floating species growing in lakes and slow-flowing rivers near the shore in Europe and Western Asia, and as an invasive plant in the USA and Canada. Light-requiring plants of this species can also grow in the shade, up to about 30% of full sunlight. In this paper we present the data about the photosynthetic apparatus of sunny and shady *H. morsus-ranae* plants grown in the sun and in the shade in nature. Methods of light and transmission electron microscopy, biochemistry, chlorophyll fluorescence induction as well as the principal component analysis were used. It was found that leaves of plants growing in shade differed from those in the sun with such traits as thickness of a blade, palisade and spongy parenchyma, ultrastructure of chloroplasts, and quantum efficiency of photosynthetic electron transport, the content of chlorophylls and carotenoids, anthocyanins and phenilpropanoids. By these traits, *H. morsus-ranae* shady plants are similar with shade-bearing plants that indicates their adaptation to light intensity lowering. The ordination plots (PCA) suggested a clear structural and functional shift of plants growing in different lighting showing relationship to light changes in the natural environment. Thus, our results displayed the high phenotypic plasticity of the *H. morsus-ranae* photosynthetic apparatus, which ensures its acclimation to changing light environment and wide distribution of this species.

## 1. Introduction

*Hydrocharis morsus-ranae* L. are monocotyledonous, dioecious, stolon-rosette free-floating aquatic plants (hydrophytes) of lakes, ponds and slow-flowing rivers, growing near the shore [1]. Flowers are white, reaching almost 2 cm in diameter. Male (staminate) flowers are usually gathered in inflorescences of three, female (pistillate) flowers are solitary. Heart shaped leaves with the spongy lower side and long petioles are collected in the rosette and float on the water surface. The color of leaves is usually a light or pure green or becoming brownish black at senescence. It can vary depending on the level of sunlight or shade, and plant age. In autumn, thin stolons with large buds (turions) at the ends appear. Turions fall to the bottom of the water body, where they remain until spring. In spring, turions develop new plants [2,3,4,5]. Micromorphology of vegetative and generative organs were described by Cutter and Feldman [6], Seago et al. [7], Tsyrenova et al. [8], Efremov et al. [9]. *H. morsus-ranae* is native for Europe and Western Asia, and it widely naturalized outside its native range in the USA and Canada as an invasive species because of its significant ability to overgrow areas in a short time by vegetative and seed propagation [4,10]. In Ukraine, *H. morsus-ranae* is usual in the Polissya (northern forest) and Forest-Steppe physiographic regions, sporadically—in Steppe and on the Zakarpatska plain [11].

*H. morsus-ranae* is often associated with aeriel-aquatic plants, including: *Typha* ssp., *Phragmites australis*, *Sparganium* spp., *Butomus umbellatus*, *Sagittaria* spp., that offer protection from currents, wind, and waves. It is supposed the sanitary role of Lemna. minor, since it is sensitive to pollution and settle only in clean waters [3] (. *H. morsus-ranae* are light-requiring plants occurring and best growing at full sun light, but also at the relatively low light levels, up to about 30% of diffuse radiation incident in an open area [12].

We investigated the responses of aerial-aquatic and true water plants at the organism, cellular and molecular levels to the unfavorable changes of water regime in nature, and in the experimental conditions during more 2 decades [13,14,15]. In the last decade, coastal thickets of T. latifolia and Ph. australis have increased significantly in the river Psyol Poltava region and the Dnipro River near Venice islands, shading sunlight for light-loving *H. morsus-ranae* plants but having no an effect on their growth and development. We hypothesized that successful growth of light-requiring plants in the shade is provided by plasticity of the photosynthetic apparatus as an adaptive role of phenotypic plasticity is well known. Therefore, investigation of plasticity is important for understanding the mechanisms of plant responses to unfavorable changes in ecological factors and plant interrelations with the changing environment [16,17] especially in the modern conditions of anthropogenic pressure on the biosphere and global climate changes. To check our assumption, we performed the comparative investigation of the structure and functional state of the photosynthetic apparatus in plants growing in the full sunlight and in the shade, the results of which are presented in this article.

## 2. Results

### 2.1. Leaf Micromorphology

**Figure 1:**
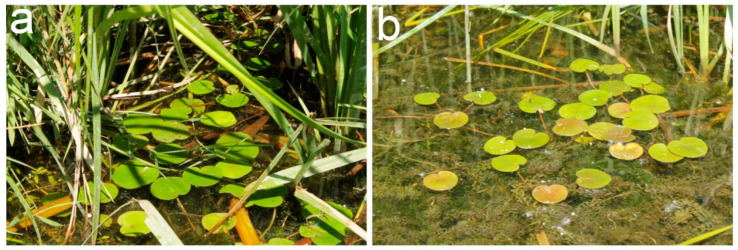
Plants of *Hydrocharis morsus-ranae* in the shade of *Phragmites australis* (**a**) and in the sun (**b**) in the wide part of the arm of the Dnipro River near the Venetian Island.

The leaves of plants, which grew in the shade had a green color (Figure 1a), while the same of plants, which grew in the full sun light, were frequently characterized by a brownish color of the adaxial surface (Figure 1b).

**Figure 2:**
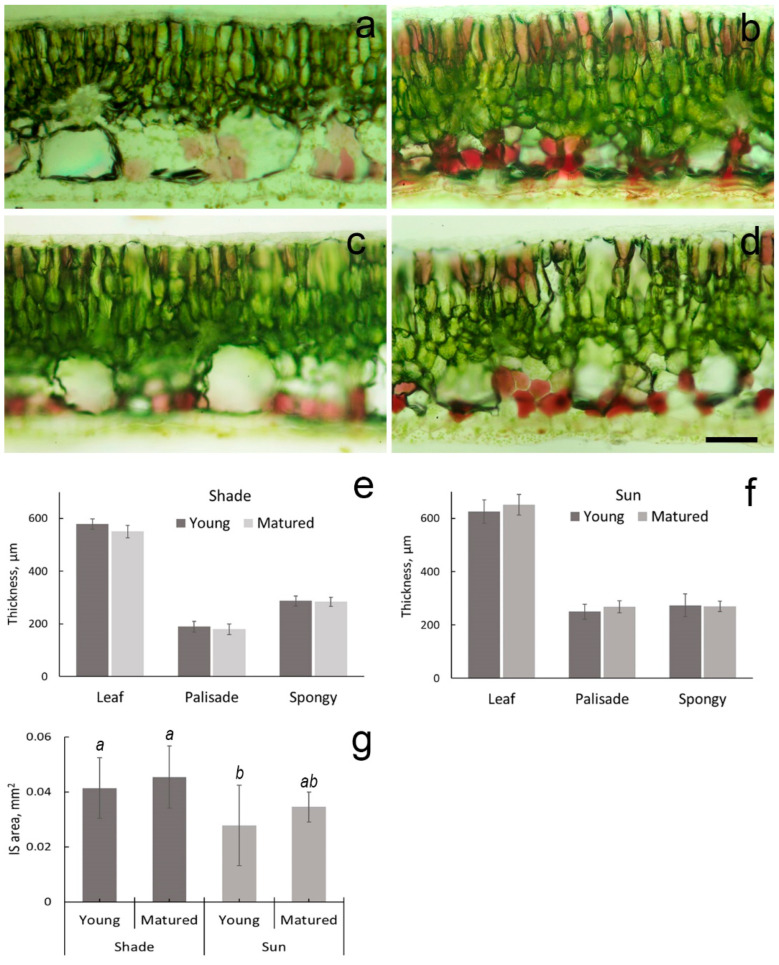
Hand-made cross-sections of *Hydrocharis morsus-ranae* young (**a**,**b**) and mature (**c**,**d**) leaves in the shade (**a**,**c**) and in the sun (**b**,**d**) without staining. Anthocyanin spots are seen in cells of the subepidermal layer of adaxial and abaxial leaf surfaces. Histograms of a leaf blade, palisade and spongy parenchyma thickness (**e**,**f**) and area of intercellular spaces (IS) in the spongy parenchyma (**g**). Different letters on Figure 2g indicate significant difference in IS area (one-way ANOVA, *p* < 0.05). Scale bar—100 µm.

Young leaves had also brownish color regardless of the location. Anatomical structure of leaves was similar in the sun and shade. Leaves were dorsoventral with a single-layer epidermis. Anthocyanins were detected in the subepidermal cell layer of abaxial and adaxial leaf sides and much more expressed in leaves facing the sun (Figure 2a–d). Palisade parenchyma consisted of two, often three layers of elongated tightly packed cells, spongy parenchyma—of four layers of loosely packed irregular or rounded cells. The thickness of a leaf blade, palisade and spongy parenchyma in young leaves in the sun and shade did not essentially differ and was 611 ± 44.2, 250 ± 28.0 and 273 ± 43.0 in leaves in the sun and 579 ± 19.6, 189 ± 19.9 and 287 ± 18.2 in leaves in the shade, respectively. At the same time the thickness of leaf blades was larger in the sun, e.g., in mature leaves 650 ± 39.2 µm and 541 ± 24.0 µm in the sun and shade, respectively, mainly due to increased thickness of palisade parenchyma cells (Figure 2e,f). In the shade, the spongy parenchyma dominated palisade in thickness, e.g., in mature leaves, the thickness of palisade parenchyma was 179 ± 19.9 µm and spongy parenchyma 284 ± 17.1 µm, in the sun, 268 ± 22.6 µm and 270 ± 19.2 µm, respectively (Figure 2e,f). The presence of large intercellular spaces was a characteristic feature of the spongy parenchyma in all leaves, but their area was less in leaves in the sun (Figure 2g).

### 2.2. Chloroplasts in the Palisade Parenchyma Cells

**Figure 3:**
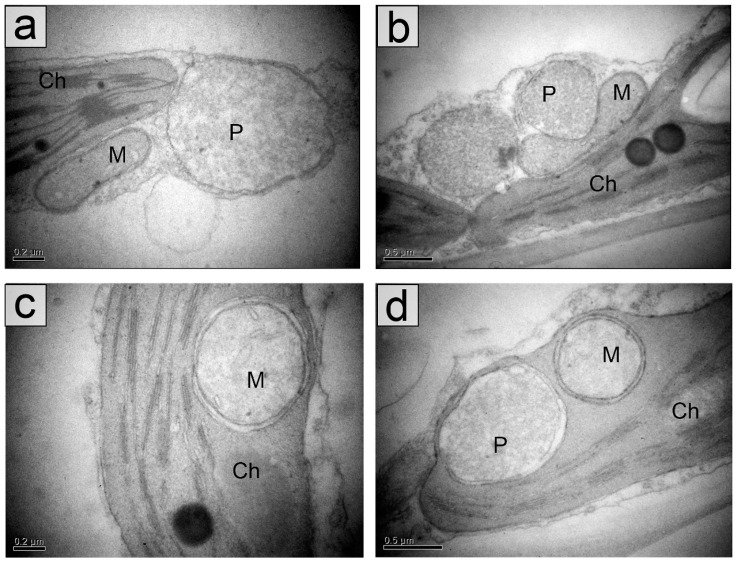
Fragments of palisade parenchyma cells of mature leaves of *Hydrocharis morsus-ranae* in the shade (**a**) and in the sun (**b**–**d**). Aggregations of chloroplasts, mitochondria and peroxisomes (**a**,**b**). Mitochondria and a peroxisome in chloroplasts’ “pockets” (**c**,**d**). Abbreviations: Ch–chloroplast, M–mitochondrium, P–peroxisome. Scale bar: (**a**,**c**) 0.2 µm, (**b**,**d**) 0.5 µm.

The ultrastructure of palisade parenchyma cells in young and mature leaves in the shade and in the sun was typical for cell such type. The main volume of cells is occupied by a central vacuole, a nucleus and other organelles are in the cytoplasm peripheral layer. Oval or elongated lens-like chloroplasts were in a contact with mitochondria and peroxisomes (Figure 3).

**Figure 4:**
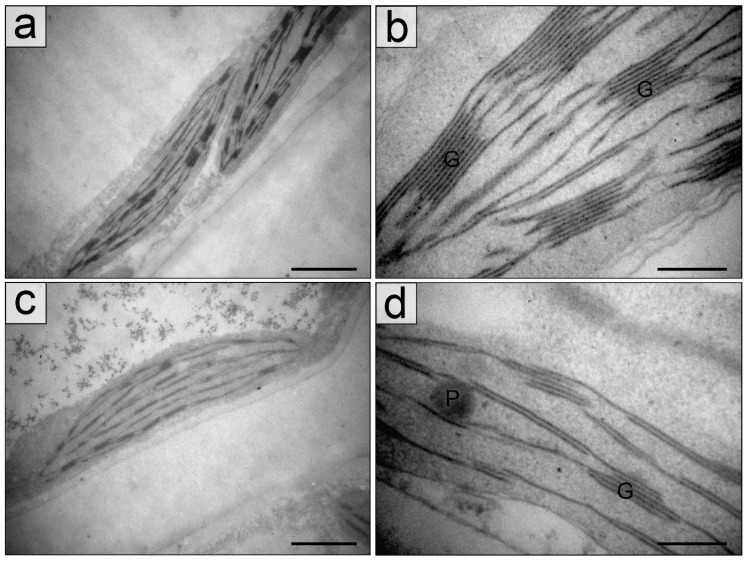
General view of chloroplasts (**a**,**c**) and chloroplast fragments with grana consisting of different number of thylakoids (**b**,**d**) from palisade parenchyma cells of young leaves of *Hydrocharis morsus-ranae* in the shade (**a**,**b**) and in the sun (**c**,**d**). Abbreviations: G—granum. Scale bar: (**a**,**c**)—1 µm, (**b**,**d**)—0.2 nm.

**Figure 5:**
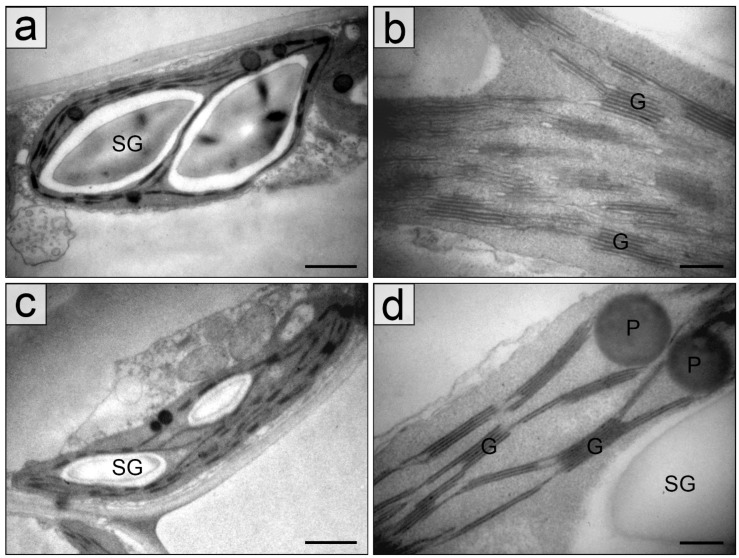
General view of chloroplasts with grana, starch grains and plastoglobules (**a**,**c**) and chloroplast fragments with grana consisting of different number of thylakoids (**b**,**d**) from palisade parenchyma cells of mature leaves of *Hydrocharis morsus-ranae* in the shade (**a**,**b**) and in the sun (**c**,**d**). Abbreviations: SG—starch grain, G—granum, Pl—plastoglobule. Scale bar: (**a**,**c**)—1 µm, (**b**,**d**)—0.2 nm.

The main differences between chloroplasts in the sun and in the shade consist in a size of organelles and a number of thylakoids in grana, both of which in the shade exceeded those in the sun (Table 1, Figure 4 and Figure 5).

An area of starch grains in chloroplasts of mature leaves especially was larger in the shade but the statistically validity differences were not found. A diameter of plastoglobules varied from 0.09 ± 0.023 µm in young leaves to 0.23 ± 0.067 µm in mature leaves in the shade and from 0.12 ± 0.021 µm in young leaves to 0.29 ± 0.051 µm in mature leaves in the sun.

### 2.3. Photosynthetic Pigments

The content of chlorophylls per unit of dry weight in mature leaves in the shade was twice higher than in the sun (Table 2). In contrast, young leaves in the sun contained even slightly more chlorophylls than mature ones. In the shade, the content of chlorophylls per unit of dry weight also was more than 2 times higher in mature leaves compared to young, but it did not differ in young and mature leaves in the sun (Table 2). The content of carotenoids followed the same pattern.

### 2.4. Anthocyanins and Phenylpropanoids

Similar trends were observed in the content of anthocyanins and colorless phenylpropanoids (Table 3). In young leaves in the sun, the content of phenylpropanoids was twice higher than in mature leaves. The young and mature leaves in the shade generally did not differ.

**Figure 6:**
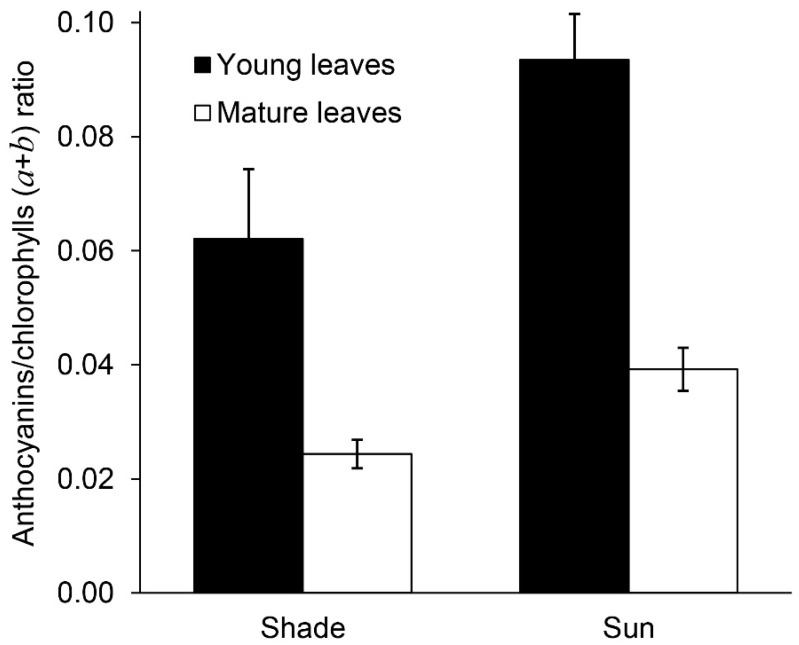
Anthocyanins/chlorophylls (*a* + *b*) ratio in young and mature leaves of *Hydrocharis morsus-ranae* in the different lighting.

A ratio of anthocyanins to the total chlorophylls decreased during leaf growth in the shade and in the sun due to an increase in the content of photosynthetic pigments (Figure 6) but it remained higher in leaves in the sun as the latter contained more anthocyanins and less chlorophylls in comparison with shady leaves.

### 2.5. Analysis of the Photosynthetic Apparatus State by Quantum Yields of Electron Transport

**Figure 7:**
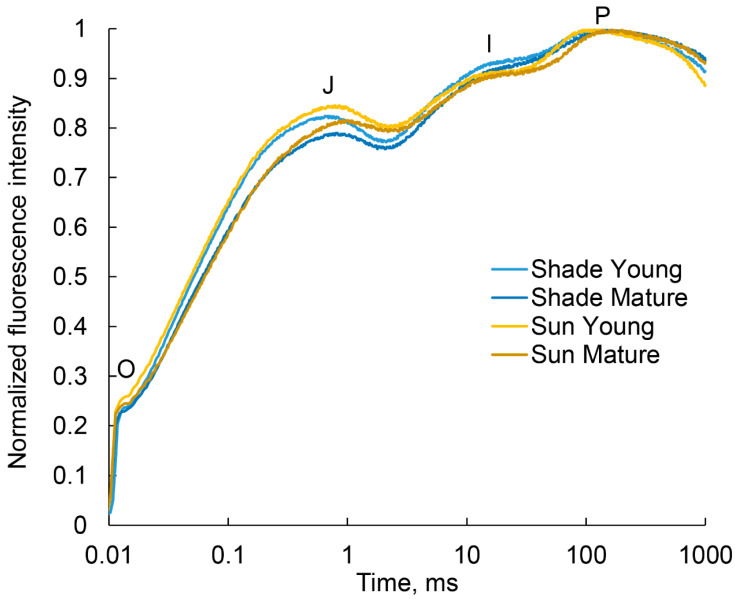
Chlorophyll a fluorescence fast induction (OJIP) averaged curves. The curves of 10 leaves for each variant were averaged and normalized to maximum.

The course of JIP fluorescence rise was essentially the same in all variants (Figure 7). The Fv/Fm parameter was some lower in young leaves in the shade than in the sun and equal in the mature leaves The Fv/Fm parameter was some lower in young leaves in the shade than in the sun and equal in the mature leaves as well as quantum efficiency of photosynthetic electron transport was also the same (Table 4). A small, but significant increase in the efficiency of electron transfer to plastoquinone was only noted in mature leaves in the shade in comparison with young leaves.

The quantum efficiency of photosynthetic electron transport did not differ significantly in plants in the different lighting. A small, but significant increase in the efficiency of electron transfer to plastoquinone was only noted in mature leaves in the shade in comparison with young leaves.

### 2.6. The Relationship between the Content of Pigments, Anthocyanins and Color in Leaves in Different Lighting

**Figure 8:**
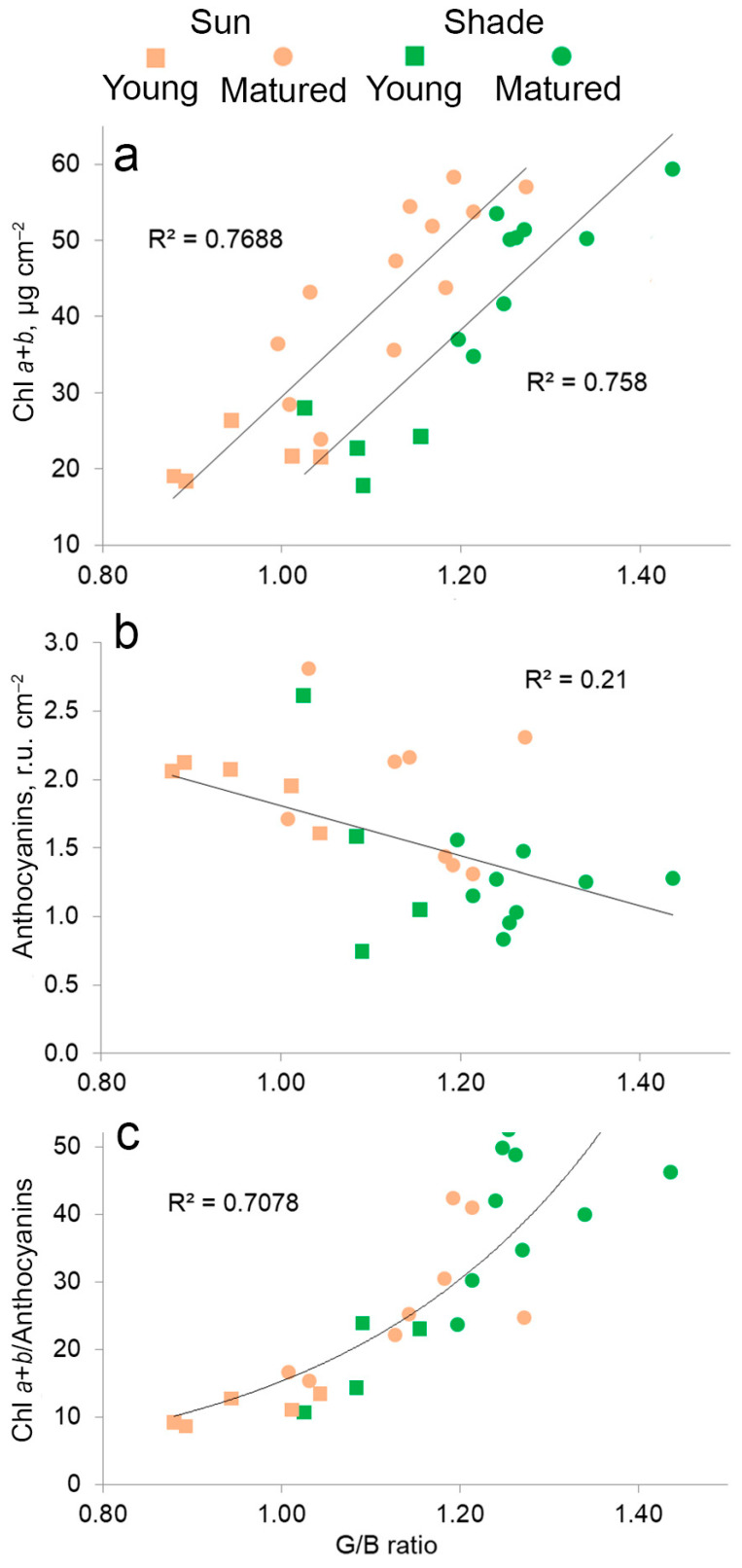
Relationship between digital values of leaf color G/B (a ratio of mean values of green to blue) and chlorophylls (**a**), anthocyanins (**b**) and ratio of chlorophylls to anthocyanins (**c**) in young and mature leaves of *Hydrocharis morsus-ranae* grown in the shade and sun.

An analysis was performed by selection of the regression models of correlation between the amounts of chlorophylls, anthocyanins and their ratio with the leaf adaxial surface color characteristic in RGB color space. Among the digital values of R, G, B, as well as their ratios R/B, R/G, G/B, (R+B)/G, G/(B*R), (R+B) * G, the G/B ratio (or B/G) was established to correlate the most strongly with the pigment content in leaves. An increase in the chlorophyll content led to an increase in the G/B ratio (Figure 8a), that allowed to find independent reliable linear models with a coefficient of determination (*r*^2^) of 0.76 and 0.75 for LL and LS, respectively, for sunny and shady leaves. At the same time, the correlation between the anthocyanins content and leaf color was weak, *r*^2^ for the combined model was 0.21 (Figure 8b). The ratio of chlorophylls to anthocyanins showed the correlation with the G/B ratio (*r*^2^ = 0.71), but for the exponential model (Figure 8c). In general, the G/B ratio in the RGB model is visualized as green–blue tints. Thus, the main factor that determines the color change during leaf growth is the accumulation of chlorophylls.

### 2.7. Principal Component Analysis of the Variability of Photosynthetic Traits

**Figure 9:**
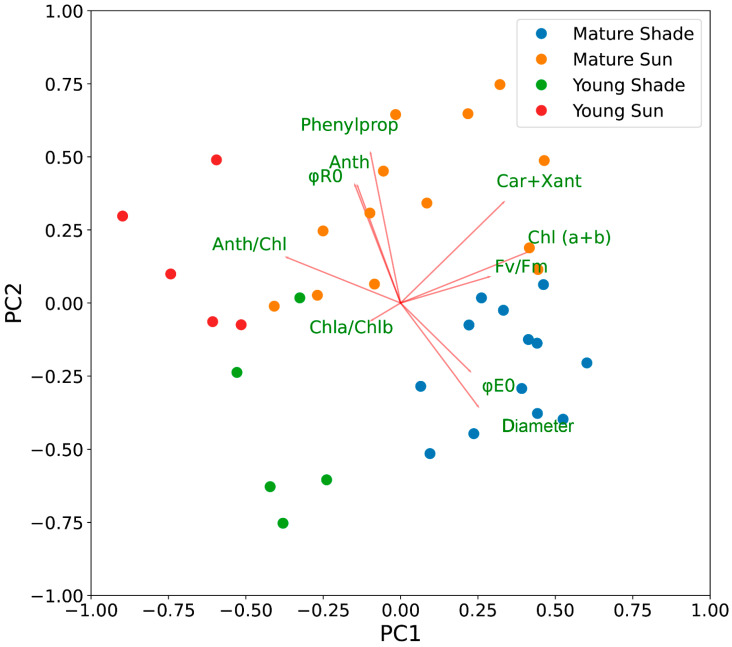
Principal component analysis (PCA) biplot of studied photosynthetic traits in leaves of *H. morsus-ranae*. The loadings of physiological traits are shown with red lines and labelled by green captions. The first principal component (PC1) is mainly associated with Chl (*a* + *b*) and F_v_/F_m_, whereas the second component (PC2) is associated with the content of phenylpropanoids, anthocyanins, and with ϕR0. The locations of leaves in the ordination space are shown by circles filled with different colors, according to the experimental group, as also indicated in the legend: blue circles—mature leaves in the shade; orange—mature leaves in the sun; green—young leaves in the shade; red—young leaves in the sun. The groups are clearly separated on the ordination plot, indicating that they are different by multivariable traits. The leaf groups are separated along with PC1 by their development stage (young and mature ones), and along with PC2 by their lighting conditions (sun and shade ones), which indicates that these factors have different and independent effect on leaves.

With principal components analysis (PCA) it was found that the first principal component (PC 1) explains 43% and the second (PC 2)—23% of all the data variance. PC 1 is primarily associated with the content of photosynthetic pigments and the chlorophyll fluorescence parameter Fv/Fm, PC 2—with the content of phenylpropanoids, anthocyanins and quantum efficiency of electron transport to the terminal acceptor (Figure 9). Interestingly, the content of carotenoids was equally strongly associated with both PC 1 and PC 2, i.e., it decreased in leaves with immature photosynthetic apparatus or in shady plants.

**Figure 10:**
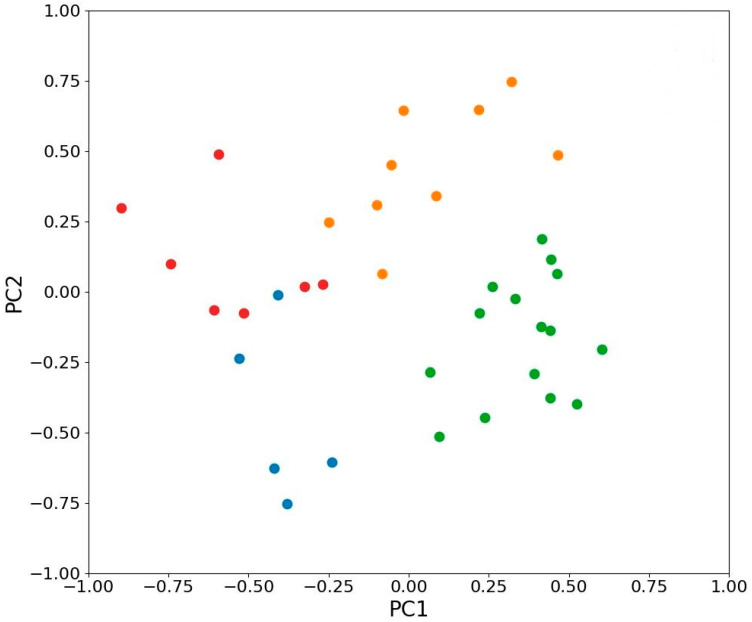
K-means clustering of PCA representations of studied leaves of *H. morsus-ranae.* The leaves were automatically assigned to different clusters (shown in different colors), that are similar to groups by their age and lighting conditions.

In the ordination plot, all leaves are clearly separated into four groups: (1) young leaves in the sun, (2) young leaves in the shade, (3) mature leaves in the sun, and (4) mature leaves in the shade (Figure 8). The distance between individual leaves in the 2-D ordination space shows their dissimilarity by a studied multivariable complex of traits. Therefore, spatial separation of different experimental groups confirms that they are substantially and consistently different. By the developmental stage, the leaves are separated along with the PC 1 axis (Figure 10), which is associated with the chlorophyll content and the level of PSII repair after photodamage. By lighting conditions, the leaves are separated along with the PC 2 axis, which is associated with the content of protective compounds—phenylpropanoids and anthocyanins, as well as with the actual efficiency of photosynthesis, suggesting their role in the protection from excessive light. Therefore, the development stage and lighting conditions have different and independent effects on the leaves.

Clustering of the leaves’ multivariate traits the by k-means algorithm [18] showed their similar grouping to our experimental one by leaf age and lighting conditions (Figure 9), also confirming that these groups are different.

## 3. Discussion

Phenotypic plasticity, which is a fundamental property of all living organisms, is defined as a genome ability to change its expression and be realized in different phenotypes in an adaptive response to various environmental influences. Phenotypic manifestations of changes in gene expression are already defined at the level of transcription efficiency and include a very broad spectrum of ecologically important traits—physiological, biochemical, anatomical and morphological, peculiarities of developmental biology, time of transferring to the reproductive stage. It is postulated that phenotypic plasticity is carried out within the limits of the normal response on the basis of metabolic, hormonal, and epigenetic regulation of gene expression and provides two strategies of the adaptation process: (1) rapid adaptation (acclimation) in response to daily and seasonal fluctuations of environmental factors and (2) long-term adaptation (acclimatization) to moderate chronic effects of adverse environmental changes factors often resulting in the appearance of a new ecotype [16,17,19,20,21,22]. The structure of the photosynthetic apparatus during adaptation to seasonal variations in the external environment, in particular lighting, has been referred by A.D. Bradshow [23] to phenotypic plasticity in the most detailed review of the known manifestations of this phenomenon at the population and species levels at that time. Now, it is considered short-term reactions to changing light intensity within seconds to minutes, and long-term adjustments over hours and days. The mechanisms of long-term acclimation to light intensity have been usually studied by changing constant light intensity. However, much less attention has been paid to photosynthesis responses to highly fluctuating light intensity in natural environments, caused by seasonal and daily periodicity and short-term clouding or leaf movement [24,25,26,27]. To understand the ability of *H. morsus-ranae* light-requiring plants to acclimate photosynthetically to shade conditions in nature, we investigated micromorphology, chloroplast ultrastructure, pigment amounts, chlorophyll a/b ratio, and photosynthetic capacity, in particular the chlorophyll fluorescence parameter Fv/Fm, of young and mature leaves in plants growing in the sunlight and in the shade. The Fv/Fm value is a robust photosynthetic parameter to indicate light quality acclimation [28].

Our results firstly showed the plasticity and coordinated changes in structural and functional traits, which allow *H. morsus ranae* light-requiring plants to shading, as it has been reported for other plants [27,29,30,31]. Leaves of *H. morsus-ranae* plants growing in the shade differed from leaves in the sun by a thinner blade and a changed ratio of the palisade and spongy parenchyma thickness. Chloroplasts in leaves in the shade distinguished first by a larger size, the presence of larger thick grana, the higher chlorophylls content, and quantum efficiency of photosynthetic electron transport. So, by these traits the *H. morsus-ranae* shady plants may be related to shade-adapted plants, the characteristic features of which are leaf blade thinning and increasing of chloroplasts’ size, a number of thylakoids in grana, and the chlorophyll content in comparison with light-requiring plants [32,33,34,35].

In shade, the content of chlorophylls in young leaves was more than 2 times lower than in mature. In the early stages of leaf development, the photosynthetic apparatus is not fully functional, so young leaves cannot utilize all the incident light energy that may irreversibly damage the components of the apparatus [36]. To avoid this, plants accumulate less chlorophyll and intensively degrade the existing one to facilitate PSII repair [37]. Considering almost the same chlorophylls a/b ratio, the photosynthetic electron transport chain in young and mature leaves of *H. morsus-ranae* is probably completely formed. The difference in chlorophyll content may be caused by impeded CO_2_ transport or assimilation leading to a regulatory reduction in the sizes or numbers of chloroplasts in mesophyll cells. This may represent a species-specific effect because the difference between young and mature leaves in terms of chlorophyll content greatly varies among plant species. At the same time, in the sun, the content of chlorophylls did not differ in young and mature leaves, that suggests that the incident light is also excessive for mature leaves, and the plants adapt to high light reducing light absorption by lowering the abundance of light-harvesting pigments, as one of the mechanisms to reduce photodamage [29]. The increased carotenoids content in mature leaves in the shade may be evidence of its function first of all as light-harvesting pigments ensuring an additional absorption of light energy in the blue–green spectrum region, 350–500 nm [38,39,40], as the plant pigment complex is a complicated and labile system, which promptly respond to environmental changes. In addition, the accumulation of chlorophylls was shown to mainly influence the leaf color change during its growth.

The Fv/Fm parameter, that was slightly lower in young leaves compared to mature ones, supports the idea that young leaves are probably generally more susceptible to photoinhibition due to limitations of CO_2_ assimilation [41]. A value of Fv/Fm, that characterizes PSII maximum potential effectiveness, in leaves adapted to darkness in the conditions when a quinone pool (QA) is fully oxidized, is approximately equal in many plants, independently of their habitat conditions [42]. The Fv/Fm parameter was in the range of 0.69–0.70 in young leaves, indicative of severe photoinhibition [43], and rose to the level of 0.73 ± 0.01 in mature leaves, still reflecting a moderate level of photoinhibition. In contrast to the developmental stage of the leaves, lighting conditions did not affect Fv/Fm, hence levels of PSII photodamage. The other two key photosynthetic quantum yields—φEo and φRo—reflect the maximum potential efficiency of electron transport at the acceptor side of PSII and PSI in dark adapted state [43]. These parameters are also sensitive to limitations by size and availability of the plastoquinone pool and Rubisco content and activity, respectively. These two parameters did not differ in all the leaves, probably suggesting that protective mechanisms effectively alleviate the low photosynthetic capacity in young leaves and excess light in mature ones. Such mechanisms may include lower accumulation of photosynthetic pigments, light avoidance-associated movement of chloroplasts, screening of photoradiation, scavenging of reactive oxygen species (ROS), heat dissipation of absorbed light energy in PSII, cyclic electron flow around the reaction centers of PSI, and the photorespiration [44]. Increased accumulation of antocyanins in young leaves, that was also shown in this work, may contribute to ROS scavenging [45].

The presence of anthocyanins in leaves is a characteristic feature of *H. morsus-rannae* plants growing in the shade and sun. Anthocyanins absorbing the green and yellow wavebands of light, between 500 and 600 nm, are assumed to protect chloroplasts from the photoinhibitory and photooxidative effects of strong light, as well as participate in plant responses to drought, UV-B, and heavy metals and resistance to herbivores and pathogens [46,47]. The highest content of anthocyanins in young leaves in the sun is clearly conformed with an idea of its photoprotective role, in young leaves in the sun, especially [48,49,50,51,52]. A high accumulation of phenylpropanoids in young leaves in the sun correlates with the same antocyanin content, that suggests their role in the protection of photosynthetic apparatus from excess light and UV radiation by scavenging injurious reactive oxygen species [53,54].

The application of principal component analysis [55,56] made it possible to visualize the differences in light-requiring plants growing in the sun and shade in nature, thereby emphasizing the ways in which light-loving plants adapt to unfavorable environmental shading. Out of all the leaves’ traits, the contents of phenylpropanoids and anthocyanins, and φR0, were most correlated with different lighting conditions (second principal component).

## 4. Materials and Methods

### 4.1. Plant Material

**Figure 11:**
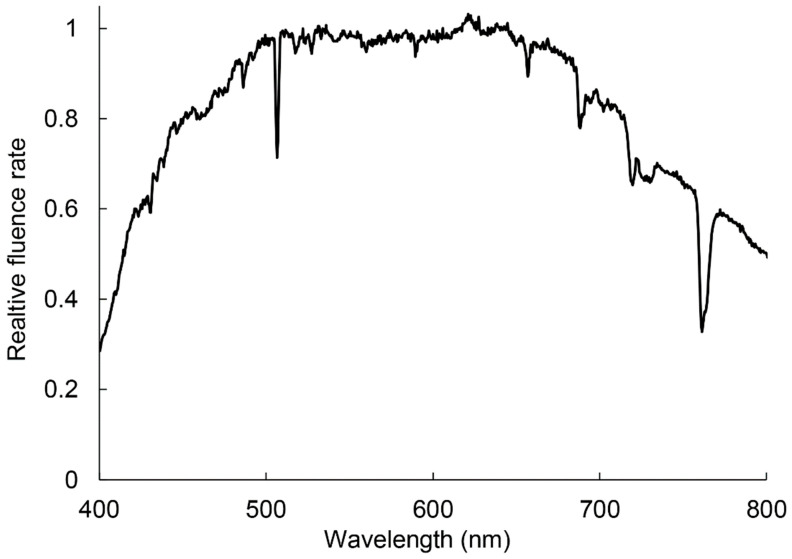
Normalized relative light spectrum distribution in shaded vs. open locations. The irradiance spectra were estimated by measurement of sunlight reflected from white etalon placed at the level of leaves.

Individual rosettes, usually containing five leaves and growing in the wide part of the arm of the Dnieper River near the Venetian Island at coordinates 50°26′37.0″ N 30°34′58.8″ in cloudless days between 10–12 am were collected. The arm is oriented in the North–South direction and is well lit most of the daytime. Plants grew on the open surface 4–6 m from the bank, where they were exposed to direct sunlight about half daylight hours, and along the shore usually between *T. latifolia* and *P. australis* plants, creating shade. The daytime average photosynthetic photons flux density (PPFD) on a cloudless day in open locations was 940 ± 80 μE·m^−2^·s^−1^, and in the shade −135 ± 70 μE·m^−2^·s^−1^, corresponding to photosynthesis-saturating and limiting light, respectively [57,58,59]. Maximum PPDF in the open locations reached 1800 μE·m^−2^·s^−1^ during plant collection. To estimate and compare the light quality, we measured reflectance of white etalon placed at the level of leaves in both shaded and open locations. The spectra were recorded, normalized by maximum value, averaged, and the spectrum of the shaded locations was divided by one in the open locations. The reflectance spectra were recorded using STS-VIS spectrometer (Ocean-Insight, Ostfildern, Germany). In shaded places, blue-green, far-red, and near-infrared parts of the spectrum were generally attenuated (Figure 11), probably causing lower light use efficiency.

The plants were transported to the laboratory within 2 h after the collection, where the measurement procedures began immediately. Young and mature leaves were analyzed separately.

### 4.2. Light Microscopy

Leaf micromorphology was studied on the hand-made uncolored sections with a light microscope Axioscope (Carl Zeiss, Oberkochen, Germany) and photographed on a digital camera EOS Rebel T2i (Canon). The thickness of the leaf blade, palisade and spongy parenchyma, and the area of intercellular spaces of the spongy parenchyma were measured on digital images of leaf cross-sections. In the diagrams, the data are presented as M ± SD, statistically significant differences between the samples were taken at *p* < 0.05.

### 4.3. Transmission Electron Microscopy

Specimen cutoffs of 3 mm diameter were fixed in 3% glutaraldehyde (0.1 M cacodylic buffer, pH 7.2) for 3 h at ambient temperature and then in 1% osmium tetraoxide in the same buffer for 1 h at ambient temperature and 12 h at 4 °C. Samples were dehydrated through a graded acetone series and embedded in epon-araldit resins. Sections were obtained on an ultramicrotome PowerTome XL (Boeckeler Instruments, Tucson, AZ, USA). Ultrathin sections (about 55 nm) were stained with uranyl acetate and lead citrate and examined with a transmission electron microscope JEM 1230EX (JEOL, Tokyo, Japan).

### 4.4. Quantification of Leaf Blade and Chloroplast Structure

A thickness of leaf blades, palisade and spongy parenchyma and a size of chloroplasts from palisade parenchyma cells of young and mature leaves were determined from TEM micrographs with program Image Tool (Version 3.0, UTHSCSA, San Antonio, TX, USA). A number of thylakoids in grana in chloroplasts on leaf cross sections was counted.

### 4.5. Leaf Color Analysis

Leaves were photographed on a white background using the EOS Rebel T2i (Canon) camera with standard optics in manual white balance mode in IPG format. Leaf color was analyzed on the obtained digital images by Eitel et al. [60] in the RGB model. The intensity average digital values of red (R), green (G) and blue (B) channels were obtained in the Image J software (Version 1.50b, https://imagej.nih.gov, accessed on 17 August 2022). In addition to absolute numerical values of channels, their different ratios (R/G, R/B, (R + B)/G, G/(R + B) and G/B were evaluated. The relationships between the pigment content and leaf color were found using R studio Software (Version 1.2.1335, https://www.rstudio.com, accessed on 17 August 2022). Regression analysis included linear and nonlinear models. The determination coefficient (*r*^2^) and the *p* value were used to assess the model reliability. Regression models with *p* values < 0.01 were considered significant.

### 4.6. Anthocyanins and Phenylpropanoids Analyses

Anthocyanins and phenylpropanoids were extracted from fresh samples (50 mg) with 0.1% HCl solution in methanol, pre-cooled to −20 °C and kept for 24 h at 2 °C and determined by the spectrophotometric method according to [61]. An analysis of the extract was performed on a spectrophotometer SF 2000 (OKB Spectr, St. Petersburg, Russia). A value of optical density at a wavelength of 532 nm, which corresponds to the maximum absorption of anthocyanins (glycoside cyanide), was corrected by subtracting 24% of the chlorophyll maximum absorption at A653. The relative amount of phenylpropanoids was evaluated by the optical density at a wavelength of 329 nm after 40-fold dilution of methanol-HCl extract.

### 4.7. Pigment Analysis

Pigments were extracted from fresh samples (50 mg) with 85% acetone. Measurements of the photosynthetic pigments content were made with a spectrophotometer SF 2000 (OKB Spectr, St. Petersburg, RF) at wavelengths of 663 nm, 647 nm and 470 nm. The content of chlorophyll and carotenoids was determined by formulas [62] and recalculated per dry mass.

### 4.8. Chlorophyll a Fluorescence Induction and JIP-Test

The state of the leaf photosynthetic apparatus was assessed with a portable OJIP fluorometer “FluorPen FP 100” (Photon Systems Instruments, Drásov, Czech Republic). This device can register polyphasic fluorescence induction curve caused by illumination of photosynthetic samples by a flash of high intensity (saturating) exciting light. The intensity of this light in our experimental setup was 5000 µmol photons·m^−2^·s^−1^. The multiple (O, J, I, P) steps of this fluorescence rise are clearly visible on the logarithmic time-axis and reflect the gradual reduction of electron carriers along the photosynthetic electron transport chain. By analyzing the parameters of this curve, it is possible to determine some traits of both light and dark phases of photosynthesis. The shape of the OJIP curve is sensitive to changes in photosynthesis caused by the environment. For the integral assessment of the photosynthetic apparatus state, the instruments sensor was pressed against the leaf blade after 15 min dark adaptation, and fluorescence changes were recorded for 1 s. Based on the obtained fluorescence curves, three key parameters, quantum yields of electron fluxes were calculated and analyzed.

1.φ. Po = FV/FM is the maximum quantum yield of the primary photochemical reaction (at t0 = 0), which characterizes the probability of energy capture of the absorbed photons (or excitons migrating by the antenna) by the reaction centers of PS 2. In the case of stress state caused by strong light or heating of the object, φPo is usually decreased.

2.φ. Eo—quantum yield of electron transfer from PS 2 to plastoquinone.

3.φ. Ro—quantum yield of reduction of electron terminal acceptors in the acceptor site of PS 1.

All calculations were performed according to Stirbet et al. [43].

### 4.9. Statistical Analysis

For exploratory analysis of the multivariate data and extraction of main leaves’ traits that constitute the differences between leaves and different experimental groups, principal component analysis (PCA) was used [56]. The degree of dissimilarity between groups by their multiple physiological traits was also assessed and visualized in 2D ordination biplot. Before PCA, the data were centered and standardized according to general recommendations.

For independent and unsupervised grouping of the leaves by their physiological traits, k-means clustering was used [18]. The number of clusters was chosen equal to the number of experimental groups, the other parameters were kept at default. For PCA and k-means clustering, and visualization of the results, python libraries ‘sklearn.decomposition.PCA’, ‘sklearn.cluster.Kmeans’, and ‘matplotlib’ were used.

For statistical analysis of significance, quantitative data have been analyzed by Student’s *t*-test and Mann–Whitney U-test.

## 5. Conclusions

A significant difference in the traits of photosynthetic apparatus was shown between *H. morsus-ranae* air-requiring plants growing in the natural environment in full sunlight and under relatively low light levels, diffuse illumination of up to 20–30% of full sunlight. The photosynthetic apparatus of plants in shade is similar to that of shade-enduring ones, which indicates its high phenotypic plasticity firstly determined by light intensity and underlying the acclimation of photosynthesis to diverse lighting conditions. The ability of light-requiring plants of *H. morsus-ranae* to adapt to low light enables their wide occurrence and survival in ponds with different lighting levels, including shadiness created by the overgrowth of higher aquatic vegetation, first of all, *P. australis* and *T. latifolia*, under climate global changes. Recently, the epigenetic system is considered pivotal in ensuring the plasticity of plant reactions to environmental signals. *H. morsus-ranae* is a suitable object to investigate the role of epigenetics in plant adaptations to habitat conditions due to its ecological patterns and successful vegetative reproduction.

## Figures and Tables

**Table 1:** Morphometric parameters of chloroplasts in palisade parenchyma cells of young and matured leaves of *Hydrochari morsus-ranae* in different lighting.

Lighting	Leaves	Chloroplast			
		Length, µm	Width, µm	Number of Thylakoids per Granum	Plastoglobule Diameter, µm
Shade	Young	9.24 ± 0.38 ^a^	1.46 ± 0.05 ^a^	6.77 ± 0.41 ^a^	0.09 ± 0.023 ^a^
	Mature	11.63 ± 0.37 ^b^	3.48 ± 0.19 ^b^	7.67 ± 0.45 ^a^	0.29 ± 0.051 ^b^
Sun	Young	6,98 ± 0,29 ^a^	1.39 ± 0.06 ^a^	4.76 ± 0.31 ^b^	0.12 ± 0.021 ^a^
	Mature	9.73 ± 0.27 ^b^	1.64 ± 0.08 ^a^	5.28 ± 0.38 ^b^	0.23 ± 0.067 ^b^

Note: there is no significant difference between the values of the parameters in columns with the same letters in uppercase at *p* ≤ 0.05, *n* = 40.

**Table 2:** The content of photosynthetic pigments in young and mature leaves of *Hydrocharis morsus-ranae* in the different lighting, mg/g dry weight ± SE.

Pigment	Shade Leaves		Sun Leaves	
	Young	Mature	Young	Mature
Chlorophyll *a*	4.09 ± 0.35 ^a^	9.95 ± 0.38 ^b^	5.12 ± 0.33 ^c^	4.82 ± 0.36 ^c^
Chlorophyll *b*	1.71 ± 0.19 ^a^	4.35 ± 0.19 ^b^	2.01 ± 0.16 ^c^	2.08 ± 0.14 ^c^
Chlorophylls (*a* + *b*)	5.79 ± 0.53 ^a^	14.29 ± 0.56 ^b^	7.13 ± 0.47 ^c^	6.90 ± 0.50 ^c^
Carotenoids	1.34 ± 0.12 ^a^	2.86 ± 0.11 ^b^	2.00 ± 0.12 ^c^	1.78 ± 0.11 ^c^

Note: in this and in Table 3 and Table 4 there is no significant difference between the values of the parameters in rows with the same letters in uppercase at *p* ≤ 0.05, *n* = 10.

**Table 3:** The content of anthocyanins and phenylpropanoids in young and mature leaves of *Hydrocharis morsus-ranae* in the different lighting, mg/g dry weight ± SE.

Substance	Shade Leaves		Sun Leaves	
	Young	Mature	Young	Mature
Anthocyanins	0.37 ± 0.10 ^a^	0.34 ± 0.03 ^a^	0.65 ± 0.03 ^b^	0.26 ± 0.03 ^a^
Phenylpropanoids	0.35 ± 0.06 ^a^	0.40 ± 0.03 ^a^	0.77 ± 0.07 ^b^	0.40 ± 0.02 ^a^

**Table 4:** Key parameters of fluorescence induction in young and mature leaves of *Hydrocharis morsus-ranae* in the different lighting.

Parameter	Shade Leaves		Sun Leaves	
	Young	Mature	Young	Mature
F_v_/F_m_	0.70 ± 0.02 ^a^	0.73 ± 0.01 ^b^	0.69 ± 0.01 ^a^	0.73 ± 0.01 ^b^
φE0	0.35 ± 0.01 ^a^	0.37 ± 0.00 ^b^	0.34 ± 0.01 ^a^	0.34 ± 0.01 ^a^
φR0	0.25 ± 0.01 ^a^	0.25 ± 0.00 ^a^	0.27 ± 0.01 ^a^	0.26 ± 0.00 ^a^

## Data Availability

Not applicable.

## References

[1] Cook C.D.K., Luond R. (1982). A revision of the genus *Hydrocharis* (Hydrocharitaceae). Aquat. Bot..

[2] Scribailo R.W., Posluszny U. (1984). The reproductive biology of *Hydrocharis morsus-ranae* L. Floral biology. Canad. J. Bot..

[3] Catling P.M., Miltrow G., Haber E., Posluszny U., Charlton W.A. (2003). The biology of Canadian weeds *Hydrocharis morsus-ranae* L.. Can. J. Plant Sci..

[4] Toma C. (2013). Reproduction of *Hydrocharis morsus-ranae* taxa in an oxbow lake of the River Vistula. Limnol. Rev..

[5] Zhu B., Ottaviani C.C., Naddafi R., Dai Z., Du D. (2018). Invasive European frogbit (*Hydrocharis morsus-ranae* L.) in North America: An updated review 2003–2016. J. Plant Ecol..

[6] Cutter E.G., Feldman L.J. (1970). Trichoblasts in Hydrocharis. I. Origin, differentiation, dimensions and growth. Am. J. Bot..

[7] Seago J.L., Marsh L.C., Stevens K.J., Soucup A., Votrubova O., Enstone D.E. (2005). A re-examination of the root cortex in wetland flowering plants with respect to aerenchyma. Ann. Bot..

[8] Tsyrenova D.J., Varfolomeeva A.S., Goroshko J.M. (2018). Micromorphology of *Hydrocharis dubia* (Blume) Backer, L. (Hydrocharitaceae) plants from the Far East of Russia.). Inland Water Biol..

[9] Efremov A.N., Grishina V.S., Toma C., Mesterhazy A., Tchatchouang E.N. (2021). Comparative morphology of the aquatic plant, genus *Hydrocharis morsus-ranae* L.. Can. J. Bot..

[10] Zhu B., Ellis M.S., Fancher K.L., Rudstam L.G. (2014). Shading as a control method for invasive European frogbit (*Hydrocharis morsus-ranae* L.). PLoS ONE.

[11] Gamulya Y.G. (2011). Plants of Ukraine.

[12] Pindel Z., Wozniak L. (1998). Natural conditions for presence of some ornamental water and peatbog plants. Agricultura.

[13] Kordyum E., Mosyakin S., Ivanenko G., Ovcharenko Y., Brykov V. (2021). Hydropotes of young and mature leaves in *Nuphar lutea* and *Nymphaea alba* (Nymphaeaceae): Formation, functions and phylogeny. Aquatic Bot..

[14] Kordyum E.L., Bluma D.A., Ivanenko G.F., Kozeko E.L., Artemenko O.A., Vedenicheva N.P. (2019). Plasticity of morphophysiological and oxidative metabolism patterns of *Sium species* (Apiaceae) at different soil moisture. J. Plant Physiol. Pathol..

[15] Kordyum E., Kozeko L., Ovcharenko Y., Brykov V. (2017). Assessment of alcohol dehydrogenase synthesis and aerenchyma formation in the tolerance of *Sium* L. species (Apiaceae) to waterlogging. Aquat. Bot..

[16] Miner B., Sultan S., Morgan S.G., Padilla D.K., Relyeae R.A. (2006). Ecological consequences of phenotypic plasticity. Trend Ecol. Evol..

[17] Dubyna D.V., Kordyum E. (2015). Ontogenesis plasticity of vascular plants: Molecular, cellular, population and cenotic aspects. Visn. Nac. Akad. Nauk Ukr..

[18] Lloyd S.P. (1982). Least squares quantization in PCM. IEEE Transact Inform Theory.

[19] Sultan S.E. (2000). Phenotypic plasticity for plant development, function and life history. Trends Plant Sci..

[20] Sultan S.E. (2010). Plant developmental responses to the environment: Eco-devo insights. Curr. Opin. Blant Biol..

[21] Kordyum E.L., Sytnik K.M., Baranenko V.V., Belyavskaya N.A., Klymchuk D.A., Nedukha E.M. (2003). Cellular Mechanisms of Plant Adaptation to Unfavourable Influences of Ecological Factors in Natural Conditions.

[22] Kelly S.A., Panhuis T.M., Stoehr A.M. (2012). Phenotypic Plasticity: Molecular Mechanisms and Adaptive Significance. Compr. Physiol..

[23] Bradshow A.D. (1965). Evolutionary significance of phenotypic plasticity in plants. Adv. Genet..

[24] Frenkel M., Bellafiore S., Rochaix J.-D., Jansson S. (2007). Hierarchy amongst photosynthetic acclimation responses for plant fitness. Physiol. Plant..

[25] Kaiser E., Morales A., Harbinson J., Kromdijk J., Heuvelink E., Marcelis L.F.M. (2015). Dynamic photosynthesis in different environmental conditions. J. Exp. Bot..

[26] Schneider T., Bolger A., Zeier J., Preiskowski S., Benes V., Trenkamp S., Usadel B., Farré E.M., Matsubara S. (2019). Fluctuating light interacts with time of day and leaf development stage to reprogram gene expression. Plant Physiol..

[27] Morales A., Kaiser E. (2020). Photosynthetic acclimation to fluctuating irradiance in plants. Front. Plant Sci..

[28] Dietzel L., Pfannschmidt T. (2008). Photosynthetic acclimation to light gradients in plant stands comes out of shade. Plant Signal. Behav..

[29] Murchie E.H., Norton P. (1997). Acclimation of photosynthesis to irradiance and spectral quality in British plant species: Chlorophyll content, photosynthetic capacity and habitat preference. Plant Cell Environ..

[30] Yano S., Terashima I. (2001). Separate localization of light signal perception for sun or shade type chloroplast and palisade tissue differentiation in *Chenopodium album*. Plant Cell Physiol..

[31] Jia H., Liggins J.R., Chow W.S. (2012). Acclimation of leaves to low light produces large grana: The origin of the predominant attractive force at work. Philos. Trans. R. Soc. Lond. B Biol. Sci..

[32] Lichtenthaler H.K., Buschmann C., Doll M., Fietz H.-J., Bach T., Kozel U., Meier D., Rahmsdorf U. (1981). Photosynthetic activity, chloroplast ultrastructure, and leaf characteristics of high-light and low-light plants and of sun and shade leaves. Photosynthesis Res..

[33] Pearcy R.W., Franceschp V.R. (1986). Photosynthetic characteristics and chloroplast ultrastructure of C3 and C4 tree species grown in high- and low-light environments. Photosynthesis Res..

[34] Boardman N.K. (2003). Comparative photosynthesis of sun and shade plants. Annu. Rev. Plant Physiol..

[35] Mathur S., Jain L., Jajoo A. (2018). Photosynthetic efficiency in sun and shade plants. Photosynthetica.

[36] Yamori W. (2016). Photosynthetic response to fluctuating environments and photoprotective strategies under abiotic stress. J. Plant Res..

[37] Tian Y., Zhong R., Wei J., Luo H., Eyal Y., Jin H., Wu L., Liang K., Li Y., Chen S. (2021). *Arabidopsis* CHLOROPHYLLASE 1 Protects young leaves from long-term photodamage by facilitating FtsH-mediated D1 degradation in photosystem II Repair. Molecular Plant.

[38] Cogdell R.J., Gardiner A.T. (1993). Functions of carotenoids in photosynthesis. Methods Enzymol..

[39] Bartley G.E., Scolnik P.A. (1995). Plant carotenoids: Pigments for photoprotection, visual attraction, and human health. Plant Cell.

[40] Sun T., Rao S., Zhou X., Li L. (2022). Plant carotenoids: Recent advances and future perspectives. Molec. Horticult..

[41] Krause G.H., Virgo A., Winter K. (1995). High susceptibility to photoinhibition of young leaves of tropical forest trees. Planta.

[42] Kržič N.Š., Gaberščik A. (2005). Photochemical efficiency of amphibious plants in an intermittent lake. Aqua. Bot..

[43] Stirbet A., Lazár D., Kromdijk J. (2018). Chlorophyll a fluorescence induction: Can just a one-second measurement be used to quantify abiotic stress responses?. Photosynthetica.

[44] Takahashi S., Badger M.R. (2011). Photoprotection in plants: A new light on photosystem II damage. Trends Plant Sci..

[45] Zhang T.-J., Chow W.S., Liu X.-T., Zhang P., Liu N., Peng C.-L. (2016). A magic red coat on the surface of young leaves: Anthocyanins distributed in trichome layer protect *Castanopsis fissa* leaves from photoinhibition. Tree Physiol..

[46] Gould K.S. (2004). Nature’s swiss army knife: The diverse protective roles of anthocyanins in leaves. J. Biomed. Biotechnol..

[47] Close D.C., Beadle C.L. (2003). The ecophysiology of foliar anthocyanin. Botanical Rev..

[48] Steyn W.J., Wand S.J.E., Holcroft D.M., Jacobs G. (2002). Anthocyanins in vegetative tissues: A proposed unified function in photoprotection. New Phytol..

[49] Manetas Y., Petropoulou Y., Psaras G.K., Drinia A. (2003). Exposed red (anthocyanic) leaves of *Quercus coccifera* display shade characteristics. Functional Plant Biol..

[50] Neill S.O., Gould K.S. (2003). Anthocyanins in leaves: Light attenuators or antioxidants?. Functional Plant Biol..

[51] Liakopoulos G., Nikolopoulos D., Klouvatou A., Vekkos K.A., Manetas Y., Karabourniotis G. (2006). The photoprotective role of epidermal anthocyanins and surface pubescence in young leaves of grapevine (*Vitis vinifera*). Ann. Bot..

[52] van den Berg A.K., Perkins T.D. (2017). Do anthocyanins function as photoprotective light screens in senescing sugar maple leaves?. J. Plant Sci. Curr. Res..

[53] Sharma A., Shahzad B., Rehman A., Bhardwaj R., Landi M., Zheng B. (2019). Response of phenylpropanoid pathway and the role of polyphenols in plants under abiotic stress. Molecules.

[54] Dong N.Q., Lin H.X. (2021). Contribution of phenylpropanoid metabolism to plant development and plant–environment interactions. J. Integrative Plant Biol..

[55] Zinoviev A.Y. (2000). Visualization of Multidimensional Data.

[56] Lever J., Krzywinski M., Altman N. (2017). Principal component analysis. Nat. Methods.

[57] Hussner A., Lösch R. (2007). Growth and photosynthesis of *Hydrocotyle ranunculoides* L. fil. in Central Europe. Flora.

[58] Chen Z.Y., Peng Z.S., Yang J., Chen W.Y., Ou-Yang Z.M. (2011). A mathematical model for describing light-response curves in *Nicotiana tabacum* L.. Photosynthetica.

[59] Lin K.-H., Shih F.-C., Huang M.-Y., Weng J.-H. (2020). Physiological characteristics of photosynthesis in yellow-green, green and dark-green chinese kale (*Brassica oleracea* L. var. Alboglabra Musil.) under varying light intensities. Plants.

[60] Eitel J.U., Vierling L.A., Long D.S., Litvak M., Eitel K.C. (2011). Simple assessment of needleleaf and broadleaf chlorophyll content using a flatbed color scanner. Can. J. For. Res..

[61] Murray J.R., Hackett W.P. (1991). Dihydroflavonol reductase activity in relation to differential anthocyanin accumulation in juvenile and mature phase *Hedera helix* L.. Plant Physiol..

[62] Lichtenthaler H.K., Buschmann C. (2001). Extraction of phtosynthetic tissues: Chlorophylls and carotenoids. Curr. Protocols Food Analyt. Chem..

